# Evaluation of the peripheral visual performance of DIMS spectacle lenses versus single vision lenses

**DOI:** 10.3389/fnins.2024.1460062

**Published:** 2024-12-03

**Authors:** Kenneth Ka King Liu, Han Yu Zhang, Daisy Ka Yan Leung, Carly Siu Yin Lam

**Affiliations:** ^1^School of Optometry, The Hong Kong Polytechnic University, Kowloon, Hong Kong SAR, China; ^2^Centre for Myopia Research, School of Optometry, The Hong Kong Polytechnic University, Kowloon, Hong Kong SAR, China; ^3^Centre for Eye and Vision Research (CEVR), Shatin, Hong Kong SAR, China; ^4^Research Centre for SHARP Vision (RCSV), The Hong Kong Polytechnic University, Kowloon, Hong Kong SAR, China; ^5^School of Medicine, Nankai University, Tianjin, China

**Keywords:** myopia control, defocus incorporated multiple segments (DIMS), perimetry, visual field, visual sensitivity

## Abstract

**Purpose:**

This study evaluates differences in the visual field performance when wearing the Defocus Incorporated Multiple Segments (DIMS) spectacle lens compared to wearing a conventional single vision (SV) spectacle lens.

**Methods:**

Twenty-one children aged 9–14 years with spherical equivalent refraction (SER) between −1.13D to −4.75D were recruited. Mid-peripheral near visual acuity (NVA) under room lighting condition (500 lux ±10%) was measured using DIMS and SV lenses, respectively. Automated static perimetry (Zeiss, Humphrey Visual Field HFA 750i) with SITA Fast 30–2 protocol was used to investigate the visual field sensitivity. During the test, the study lens (Plano DIMS or SV lens) were inserted into the lens holder in front of the trial lenses with each child’s compensated prescription.

**Results:**

Three children were not able to complete the reliable visual tests due to fixation losses (>20%) or high false positive rate (>15%) while 18 children successfully completed the test. The mean visual field sensitivity was 29.2 ± 3.7 decibels (dB) and 29.3 ± 3.5 dB when wearing DIMS and SV lens, respectively. The mean sensitivity differences between DIMS and SV lens among 76 locations ranged from −2.4 ± 3.9 dB to 1.6 ± 3.9 dB. No statistically significant difference in sensitivity was observed across 76 locations within the central 30^o^ between DIMS and SV lens (Wilcoxon signed rank test with bonferroni correction for multiple comparisons, *p* > 0.00065). Compared to SV lens, 0.05 logarithm of minimal angle of resolution (logMAR) reduction in mid-peripheral NVA in all 4 quadrants (Superior, Temporal, Inferior and nasal, *p* < 0.05) was noted with the DIMS lens (*N* = 18). However, no statistically significant correlation was found between the mid-peripheral NVA and visual sensitivity at the specific locations.

**Conclusion:**

Although the mid-peripheral NVA was slightly reduced using DIMS lens, wearing DIMS lens did not change the children’s visual sensitivity to detect the static stimulus within 30^o^ visual field when compared to wearing SV lens.

## Introduction

Myopia is one of the most common public health concerns in the world as it increases the risks of the ocular complications such as glaucoma, cataract and retinal detachment (Haarman et al., 2020). It is estimated that the prevalence of myopia is rapidly increasing with forecasts for approximately half of the world population being myopic in 2050 (Fricke et al., 2018). High myopia has been documented to be associated with ocular diseases such as glaucoma, cataract, myopic macular degeneration (Flitcroft, 2012). The risk of ocular complications raises interest in investigating the methods to hinder myopia onset and slow the myopia progression rate.

It has been demonstrated that myopic defocus could be used for retarding myopia progression in both animal and human studies (Tse et al., 2007; Walline et al., 2011; Anstice and Phillips, 2011). Using the theory of myopic defocus, many optical devices have been investigated such as, orthokeratology lenses (OK lens) (Hiraoka, 2022), multifocal soft contact lenses (Chamberlain et al., 2019; Lam et al., 2014). Defocus Incorporated Multiple Segments lenses spectacle (DIMS) which is commercially marketed as MiYOSMART spectacle lens by HOYA. The DIMS lenses were designed with a central optical zone and surrounded by multiple segments of constant myopic defocus (+3.5 D) in the mid-periphery, providing clear central vision and peripheral myopic defocus simultaneously. A 24-month randomized clinical trial (RCT) on myopia control using spectacles lenses (DIMS) reported an efficacy of 59% in slowing myopia progression and 60% in slowing axial elongation when compared with the control group of children who wore single vision lenses (Lam et al., 2020a). The myopia control effects by DIMS lenses were sustained over 3 years (Lam et al., 2021).

Several studies have been conducted to investigate the visual performance of using optical myopia control devices. It has been reported that visual and optical quality showed a reduction after ortho-k treatment (Liu et al., 2018). DISC lens showed significant improvement in visual performance and subjective acceptance in Chinese children compared with SV spectacle lenses, and such benefits provided by DISC lenses contribute to greater satisfaction than SV spectacles for myopic children (Han et al., 2022). And our previous study showed no impacts on visual functions such as the central visual acuity, lag of accommodation, and stereopsis when wearing DIMS lenses over 2 years (Lam et al., 2020b).

However, the defocus power in the peripheral area of the DIMS lenses may lead to blurring in the mid-peripheral visual field (Jaskulski et al., 2020). Such blur in mid-periphery may lead to a decrease in visual function sensitivity (Maiello et al., 2017), which also correlates with visual field loss (Hawkins et al., 2003). The loss in the visual field may impact the quality of life, for example, reduced vision when watching television or reading or increasing the risk of falling and injury (Mckean-Cowdin et al., 2007). Children may not be willing to wear the lenses long-term if their quality of life is affected. On the other hand, testing visual acuity may provide the patient’s ability to discriminate the fine details. However, it is worth investigating the ability to detect lower spatial frequency targets in the peripheral vision as it may affect the overall pattern vision (Fleishman et al., 1987). In fact, peripheral visual performance is crucial for the daily activities as it provides essential information beyond the central gaze, especially in attention-processing tasks, peripheral motion detection and visual sensitivity. It aids in monitoring the environment for potential hazards or changes, planning actions such as steering a car or navigating stairs, and guiding eye movements to areas requiring further attention. Despite delivering less detailed information than central vision, it offers a broader view that is crucial for multitasking and situational awareness. In reality, the perception of the environment with different spatial frequencies may also depends on the peripheral visual function (Vater et al., 2022). However, few research have been conducted to study the peripheral visual performance of optical myopia control devices using myopic defocus theory. Therefore the current study aims to evaluate the visual field performance when wearing DIMS spectacle lenses.

## Materials and methods

Children aged 9–14 were recruited as they are the majority population to benefit from the myopia control lenses and the 24 months randomized clinical trial (RCT) on myopia control using DIMS lens was conducted in children in this age group (Lam et al., 2020a). The inclusion criteria were as follows: (1) Spherical equivalent refraction (SER): −1.00 to −6.00 Dioptres (D); (2) Astigmatism and anisometropia of 1.50D or less; (3) Monocular best-corrected visual acuity (VA) is equal to or better than 0.00 logarithm of minimal angle of resolution (logMAR); (5) Acceptance of the masked study design; (6) No ocular pathology.

Twenty one myopic schoolchildren (9 males and 12 females) were enrolled in the study. All procedures of the study followed the tenets of the Declaration of Helsinki and was approved by the Human Subjects Ethics Committee of the Hong Kong Polytechnic University Institutional Review Board (Ref no.: HSEARS20221201001). Informed parental consent and children’s assent were obtained before conducting the measurements.

After a comprehensive ocular health examination, cycloplegic eye drops (One drop of proparacaine 0.5% and then one drop of cyclopentolate HCL 1%) were instilled into the eyes and tested to confirm cycloplegia. Subjective refraction with maximum plus for best corrected VA was performed to determine the prescription. The spectacles lenses (DIMS and Single Vision) were ordered based on the cycloplegic subjective refraction. A randomization was done using Excel spreadsheet to determine which type of the lenses that subject wear first. Children were asked to wear the spectacles (DIMS or Single Vision) for 1 week. At the end of week, children were asked to return to the research clinic to swap their spectacles to the other option. Children were then asked to return to the research clinic at the end of the second week to have their mid-peripheral near VA tested.

**Figure 1:**
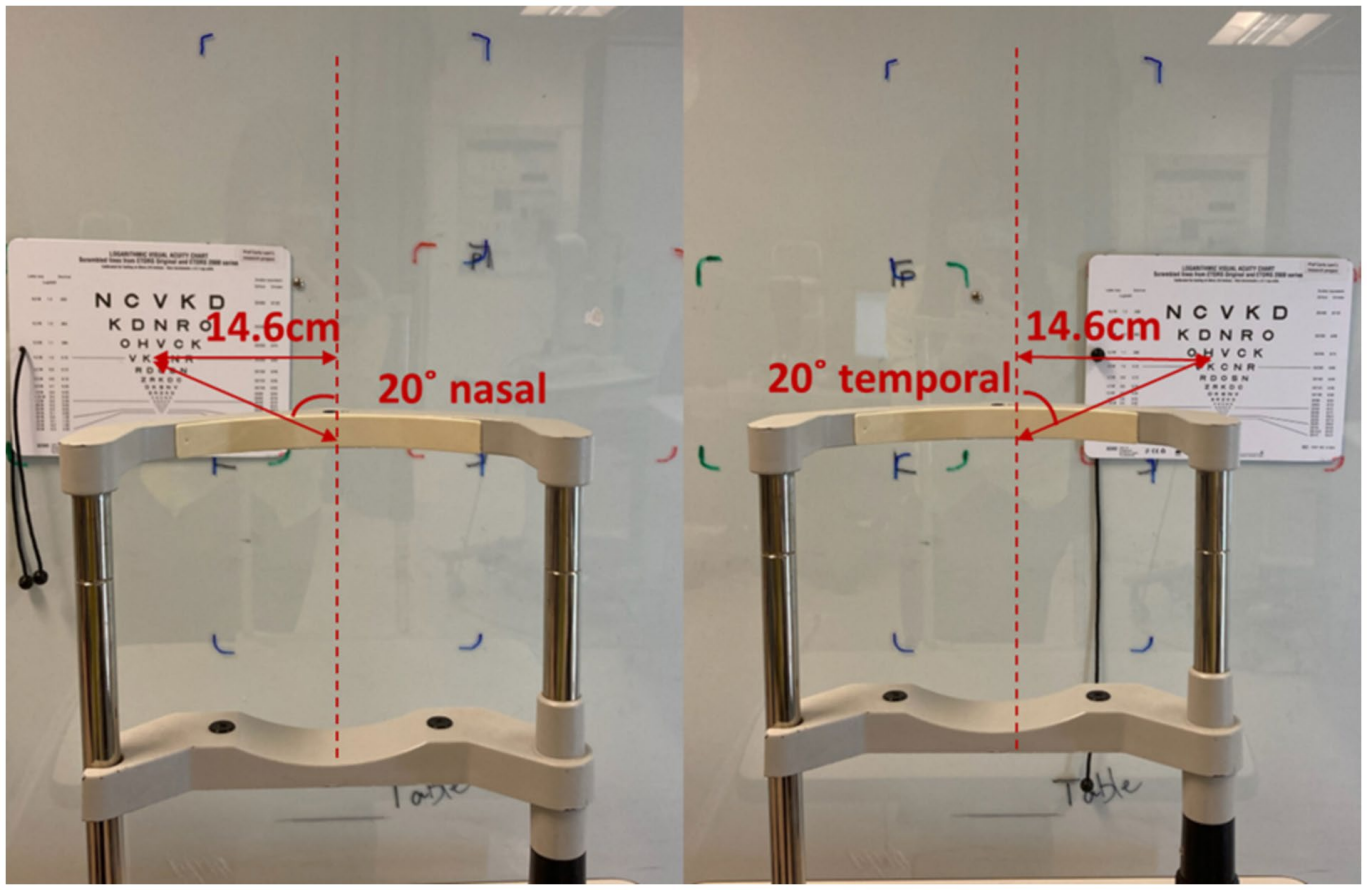
High contrast horizontal peripheral NVA in room lighting.

**Figure 2:**
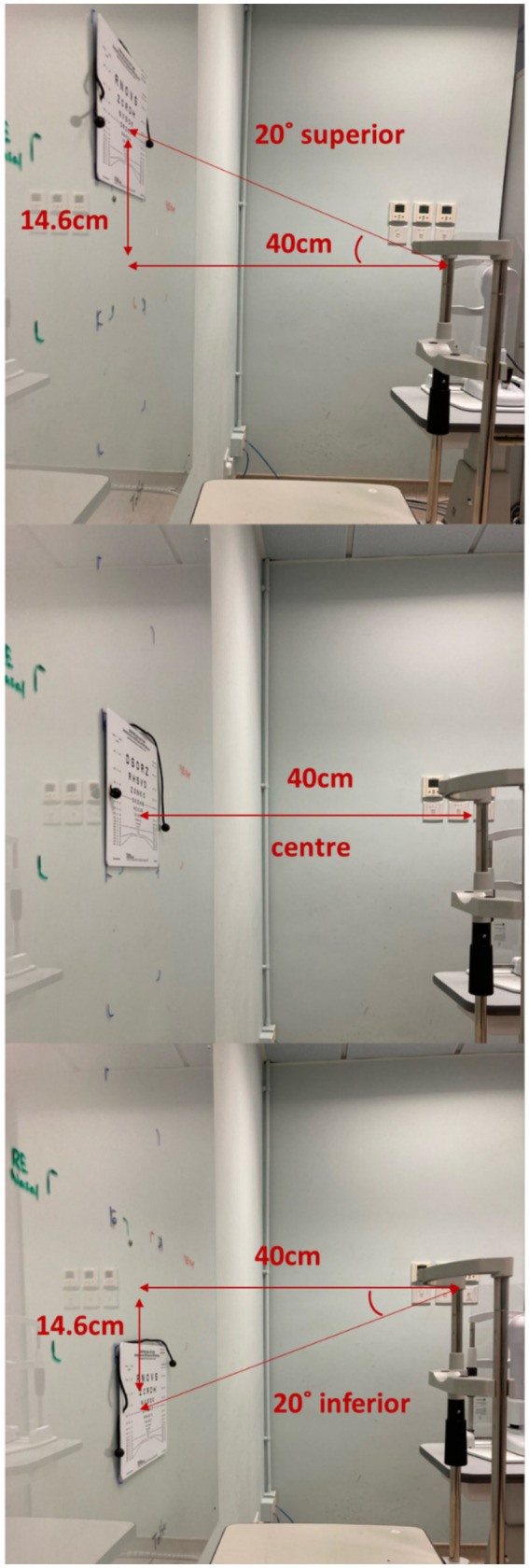
High contrast vertical peripheral NVA in room lighting.

Near visual acuity (NVA) through the mid-peripheral zone of the lens was assessed using a high contrast (100%) near visual acuity chart under room lighting (500 lux ±10%). To ensure the subject was looking through the mid-peripheral zone of the lens, the subject’s head was stabilized using a chin rest and the Near Visual Acuity chart was placed 20 degree off central visual axis superiorly, temporally, inferiorly and nasally (Lu et al., 2019; Figures 1, 2). The working distance of near visual acuity chart was set at 40 cm (Figure 2).

**Figure 3:**
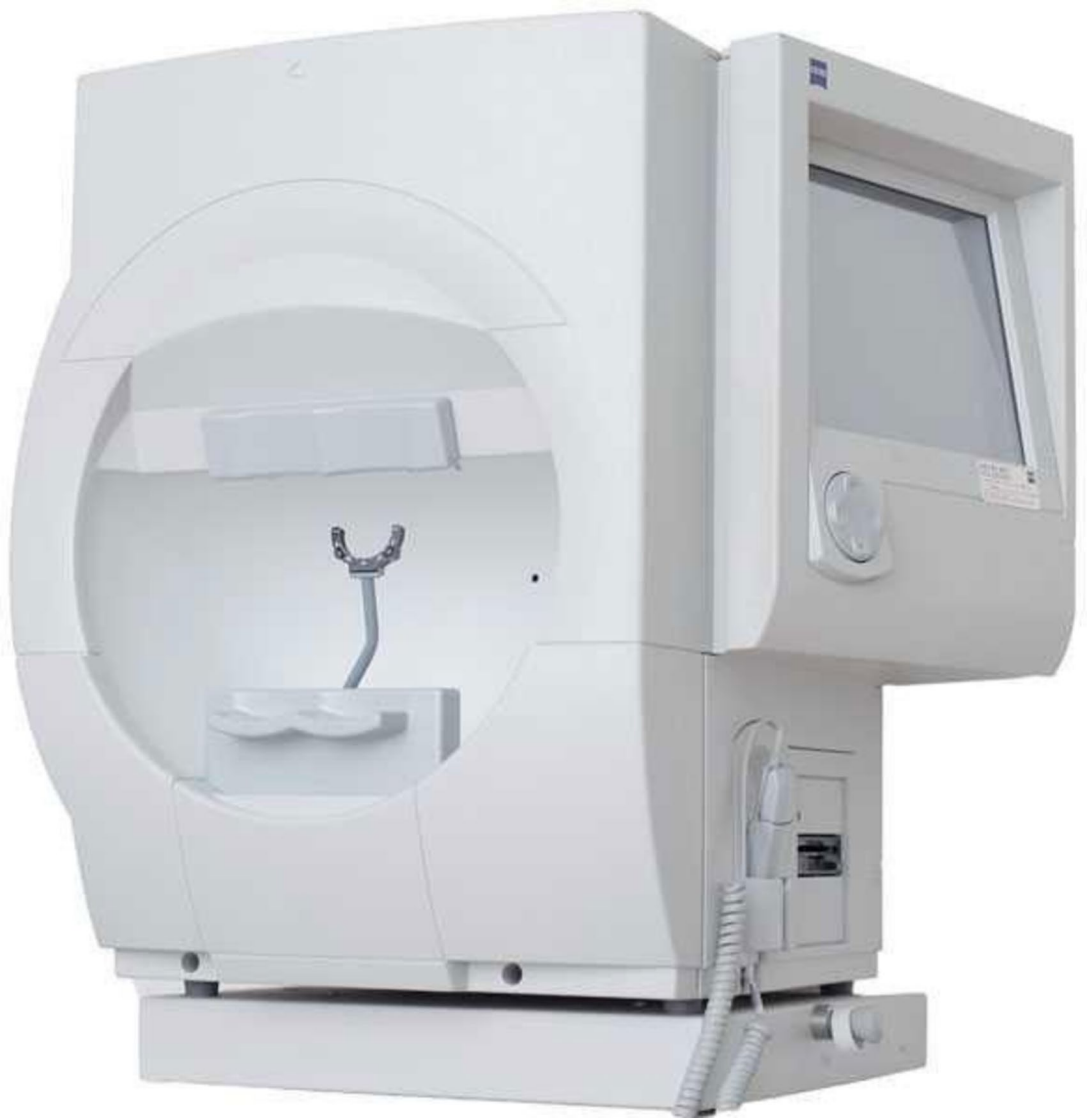
The Zeiss, Humphrey Visual Field HFA 750i visual field analyzer.

Children were asked to attend on a different day for the visual field test. The visual field test was completed using a refractive correction calculated by the visual field machine (Zeiss, Humphrey Visual Field HFA 750i) combined with a randomly selected plano test lens (DIMS or SV). The visual field was assessed using the Zeiss, Humphrey Visual Field HFA 750i analyzer, SITA Fast III, central 30–2 threshold (Goldmann III, size 0.43^o^) examination function. Table 1 shows the study flow chart of the visual field test. Figure 3 shows the visual field machine.

**Table 1:** Study flow chart for the visual field test.

Step	Procedure
1	Subject’s subjective refraction under cycloplegia was input into the visual field machine
2	The visual field machine generates a compensated prescription for each subject
3	A plano test lens (DIMS or Single vision lens) was used based on the sequences of the spectacles wear (If the subject wear DIMS spectacle lenses first, then a plano DIMS lens was used at the beginning).
4	The test lens and compensated prescription lenses were inserted into the lens holder inside the visual field machine
5	Select the protocol: Central 30–2 threshold, SITA FAST III, Goldmann III, size 0.43°
6	A plastic eye shield was used to occlude the left eye
7	Verbal instructions relating to performance of the visual field examination were given to the subject before conducting the test
8	Fixation efficiency was monitored by the investigator. If the fixation loss is more than 20% in the first trial, the investigator could interrupt the test to reposition and re-instruct the subject about the system before the examination was re-started. However, if the fixation loss was still more than 20% in the third trial, the test was kept ongoing.
9	The results of the visual field test were printed out automatically from the machine.
10	A 5-min break was given between testing each type of the lens

Using a “video game” approach, the following instructions were given to the participants:

1. The subjects were asked to look into the steady orange light of the center of a bowl-shaped instrument, which is called a perimeter.
2. The untested, fellow eye (left eye) was covered with a patch. The tested eye (right eye) was fully corrected with a trial lens and two tested lenses (SV and DIMS) were inserted in a random order in front of the right eye.
3. Subjects were asked to keep looking at a center target throughout the test. Small, dim lights appeared in different places throughout the bowl, and subjects needed to press a button whenever they were aware of the light. The machine tracks which lights the subjects did not see.
4. The subjects were allowed to blink normally during the test.
5. The light blinks at each location with differing amounts of brightness, and the machine detects the dimmest light the participants can see at each location in the peripheral vision.
6. Fixation efficiency was monitored by the investigator. If the fixation loss is more than 20% in the first trial, the investigator could interrupt the test to reposition and re-instruct the subject about the system before the examination was re-started. However, if the fixation loss was still more than 20% in the third trial, the test was kept ongoing.
7. The results of the visual field test were printed out automatically from the machine.

### Study lenses

**Figure 4:**
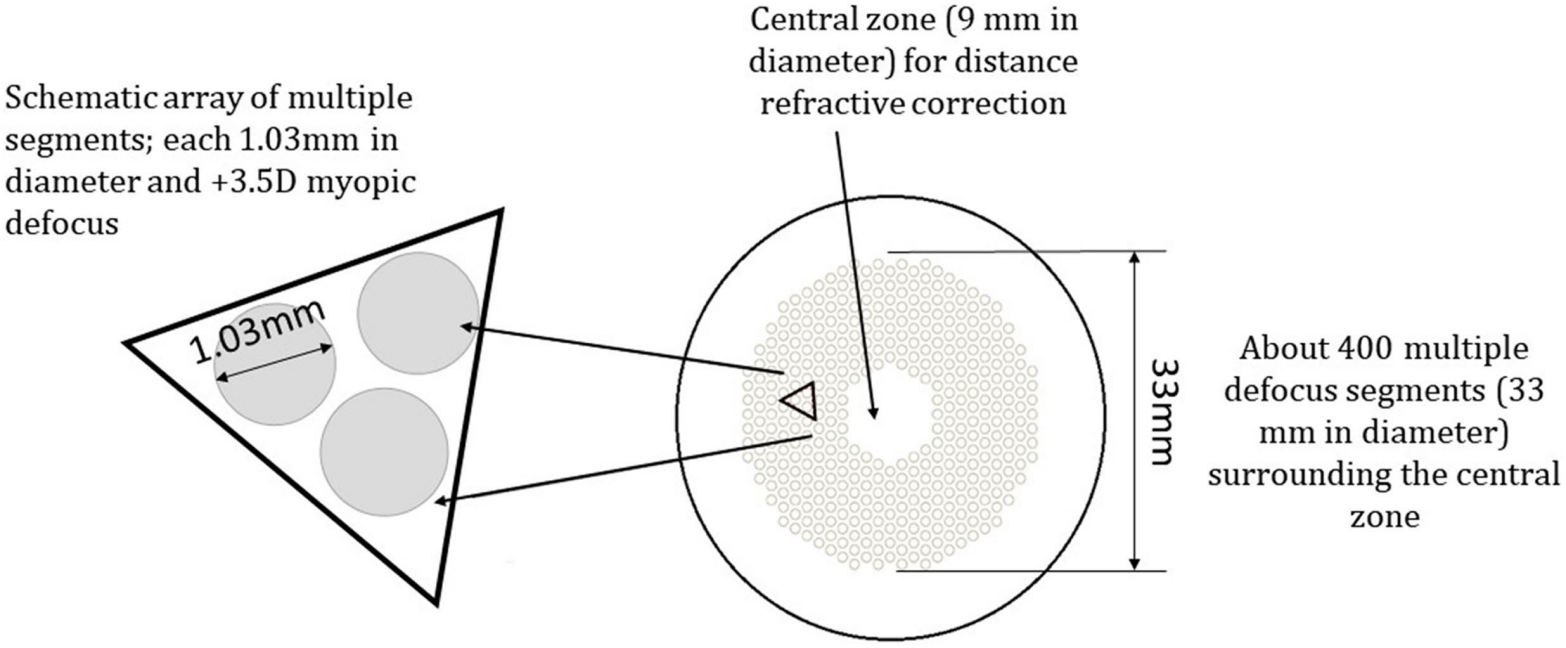
The design of the defocus incorporated multiple segments lenses (DIMS) (Lam et al., 2020a).

**Figure 5:**
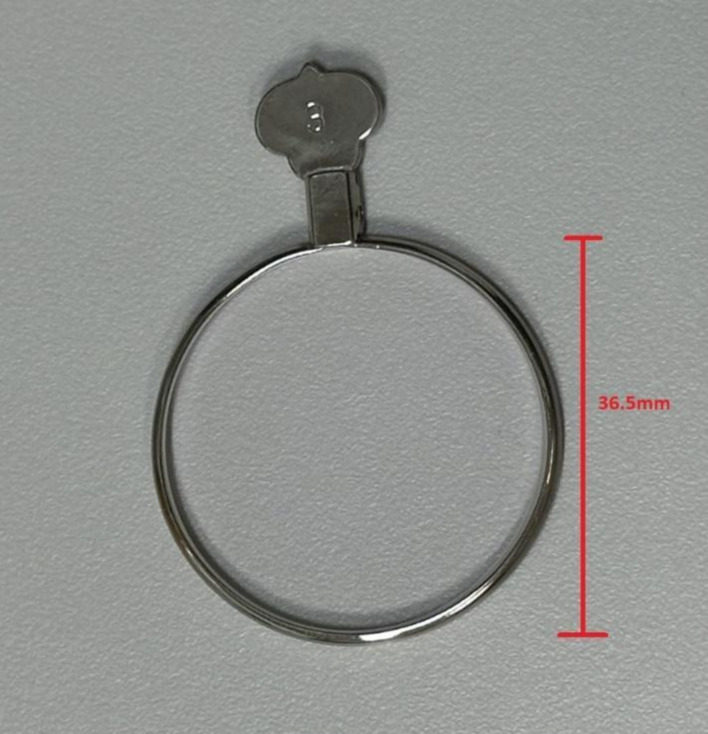
The edged DIMS fitted in to a 36.5 mm diameter trial lens ring.

The DIMS spectacle lens is a myopia control lens that has a proven efficacy in slowing myopia progression (Lam et al., 2020a; Lam et al., 2021). It is made of a central optical zone (9 mm in diameter) for distance correction of refractive error with multiple defocus segments (33 mm in diameter). Each segment with a 1.03 mm diameter contains +3.5 D defocus power (Figure 4; Lam et al., 2020a). A plano DIMS lens was edged into the 36 mm trial lens ring (Figure 5) in order to fit the trial lens holder of the visual field machine (Figure 3). The edged DIMS lens was centered to ensure the central optical zone and defocus segments were present. The edged DIMS lens or SV lens was positioned nearest to the subject’s eye on the lens holder, followed by the compensated prescription of each subject. The compensated prescription was calculated by the visual field machine based on the viewing distance of 30 cm, the subject’s subjective refraction and their age.

### Statistical analysis

The results of visual sensitivity were analyzed using normality test (*p* < 0.05, Kolmogorov–Smirnov test). Wilcoxon signed rank test with bonferroni correction for multiple comparisons was used to compare if there was any statistically significant differences in visual sensitivity between DIMS and SV lens. Bonferroni correction was used to adjust the *p* value for multiple comparisons, the statistical level of *p*-value <0.00065 was regarded as significant. Paired-t test was used to investigate if there was any significant difference in mid-peripheral NVA between DIMS and SV lenses under room lighting condition. The relationship between mid-peripheral NVA and visual sensitivity was detected by Pearson correlation. All statistical analyses were performed by using commercially available software SPSS v.16.0 (SPSS Inc., Chicago, IL, United States).

## Results

Eighteen children successfully completed the visual field assessments. Three children were not able to complete the visual field tests due to fixation losses (>20%) or high false positive rate (>15%). Only data from reliable visual field assessments were included in the normative data analysis ensuring true visual field sensitivity. Table 2 shows the demographic data of the subjects. There were no significant differences in the reliability parameters between the two lenses (*p* > 0.05). No significant difference was found in the test duration between DIMS and SV lens (*p* = 0.40). The duration for the visual field test was 4:08 ± 0.18 min and 4:14 ± 0.29 min for DIMS and SV lens, respectively (Table 3).

**Table 2:** Demographic data of eligible subjects.

Age (years)	Gender (male; female)	Spherical equivalent (diopters)
11.86 ± 1.83	9; 12	−3.09 ± 1.09

**Table 3:** Comparison of the reliability parameters and test durations of Zeiss Humphrey Visual Field HFA 750i SITA Fast III, central 30–2 threshold assessment using DIMS and SV lens.

	DIMS	SV	*p*
Fixation losses, % (median)	8.33	8.33	0.68
False positive, % (median)	0.50	3.50	0.10
False negative, % (median)	3.50	0.00	0.12
Test duration, minutes (mean ± Standard deviation)	4:08 ± 0.18	4:14 ± 0.29	0.40

**Figure 6:**
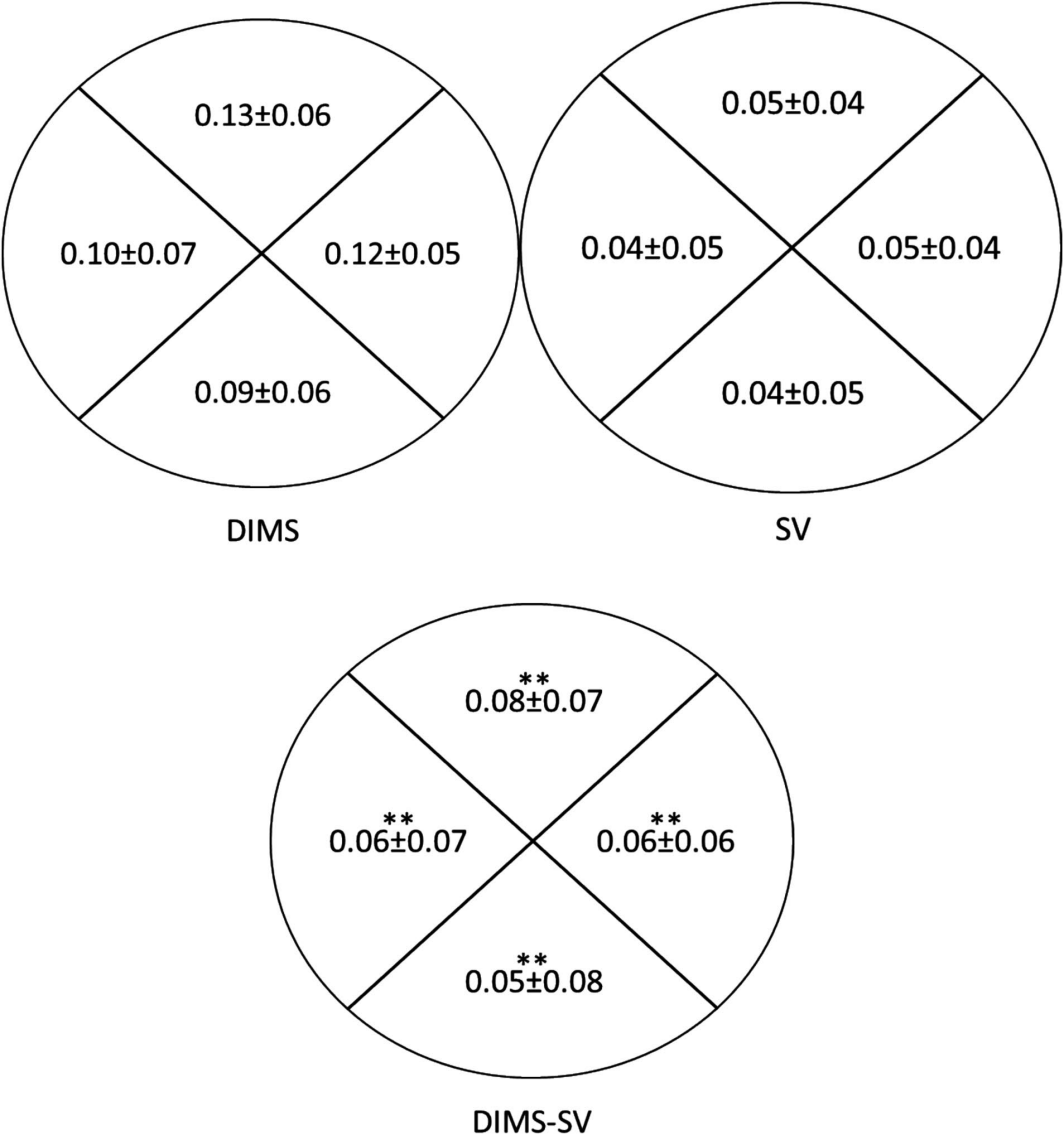
Comparison of mid-peripheral near visual acuity (NVA) under room lighting (500lux ± 10%) using DIMS and SV lenses (*N* = 18). The data presented inside the ring (Starting from the top, clockwise) indicates the mid-peripheral NVA through the superior, temporal, inferior and nasal quadrants, respectively. The NVA was expressed in logarithm of minimal angle of resolution (logMAR). The top left diagram shows the mid-peripheral NVA using DIMS lens. Top right diagram shows the mid-peripheral NVA using SV lens. The bottom diagram shows the difference in mid-peripheral NVA between DIMS and SV lenses. *p < 0.05.**p < 0.01.

Figure 6 highlights the significant differences in mid-peripheral NVA between DIMS and SV lenses in all 4 quadrants (Superior, Temporal, Inferior and nasal). On average, the mid-peripheral NVA in DIMS lens was worse than SV lenses by approximately 0.05logMAR or worse.

**Figure 7:**
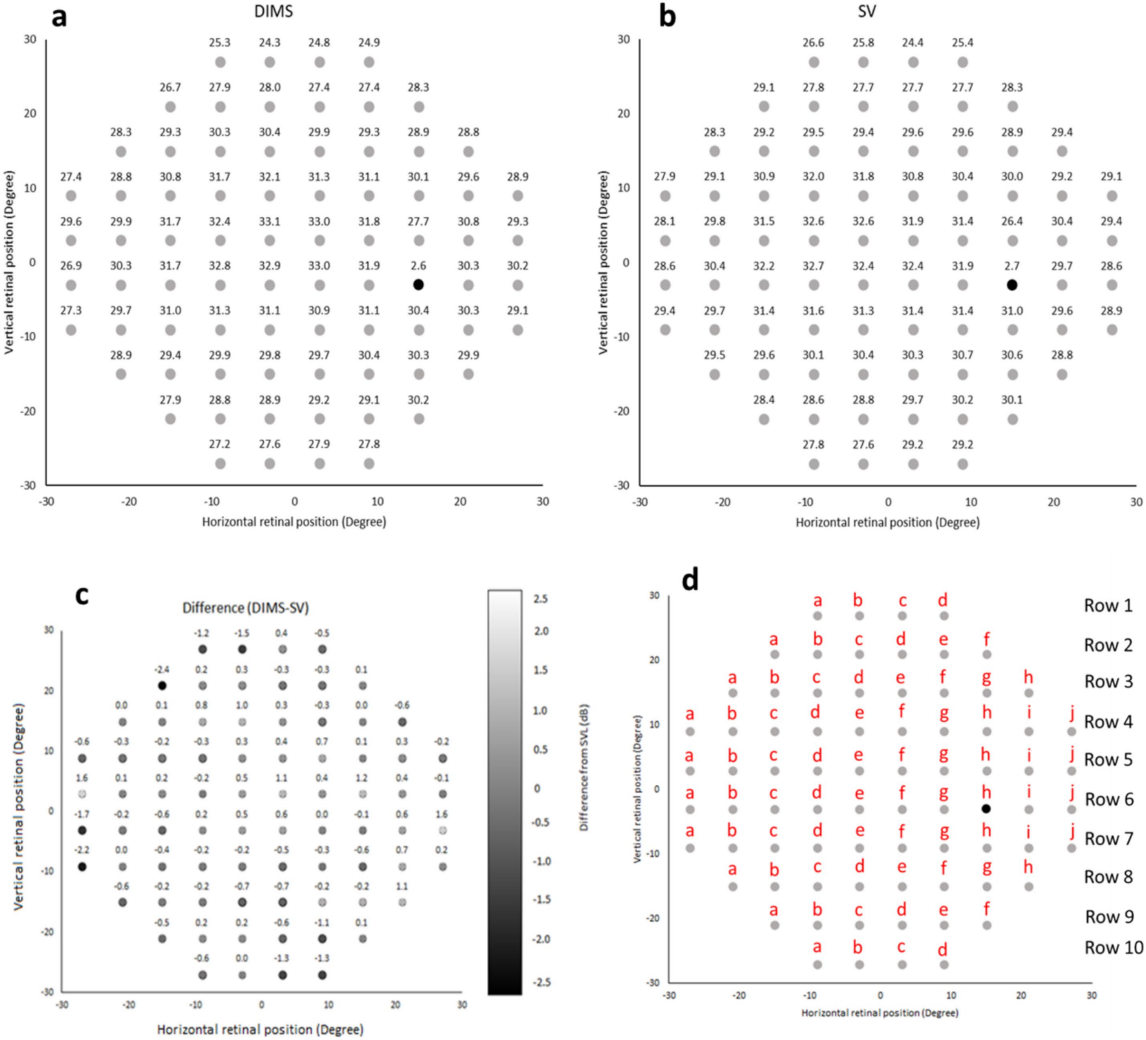
**(a,b)** Visual sensitivity map using DIMS and SV lens, respectively. **(c)** The mean difference in visual sensitivity at each retinal location within 30-degree eccentricity between DIMS and SV lenses. The positive number represents increased sensitivity using the DIMS lens while the negative number represents decreased sensitivity using the DIMS lens compared to the SV lens. **(d)** The corresponding points among the 76 locations in 30–2 SITA Fast visual field.

The mean visual field sensitivity was 29.2 ± 3.7 decibels (dB) and 29.3 ± 3.5 dB using DIMS and SV lens, respectively. The average visual sensitivity for DIMS and SV lens at each of the 76 locations are shown in Figures 7a,b. The mean difference in visual sensitivity between DIMS and SV lens is illustrated in Figure 7c. The differences ranged from −2.4 ± 3.9 dB to 1.6 ± 3.9 dB in the different visual filed locations. No statistically significant difference were found in visual sensitivity between DIMS and SV lens using Wilcoxon signed rank test with bonferroni correction for multiple comparisons (All *p* > 0.00065).

Although the mid-peripheral NVA was slightly reduced with the DIMS lens compared to SV lens (0.05–0.08 logMAR, Figure 6), there was no significant correlation between the visual sensitivity and the mid-peripheral NVA (Tables 4, 5). Figure 7d illustrates the corresponding locations. It may imply the drop in the mid-peripheral NVA may not affect children’s visual sensitivity.

**Table 4:** Correlation between horizontal peripheral NVA and visual sensitivity in SV and DIMS lenses group.

Retinal location	Peripheral NVA			
	20 degree nasal VA (SV)	20 degree temporal VA (SV)	20 degree nasal VA (DIMS)	20 degree temporal VA (DIMS)
Row 5b	−0.16 (0.53)	NA	0.02 (0.93)	NA
Row 5c	−0.13 (0.60)		0.06 (0.82)	
Row 6b	0.07 (0.79)		0.06 (0.80)	
Row 6c	0.05 (0.86)		0.17 (0.50)	
Row 5i	NA	−0.34 (0.16)	NA	0.17 (0.51)
Row 6i		−0.71 (0.001)		0.60 (0.01)

Coefficient (*p*-value). *p < 0.00065.

**Table 5:** Correlation between vertical peripheral NVA and visual sensitivity in SV and DIMS lenses group.

Retinal location	Peripheral NVA			
	20 degree superior VA (SV)	20 degree inferior VA (SV)	20 degree superior VA (DIMS)	20 degree inferior VA (DIMS)
Row 2c	−0.39 (0.11)	NA	0.04 (0.87)	NA
Row 2d	−0.21 (0.41)		0.35 (0.16)	
Row 9c	NA	−0.43 (0.07)	NA	0.09 (0.74)
Row 9d		−0.49 (0.04)		−0.11 (0.67)

Coefficient (*p*-value). *p < 0.00065.

## Discussion

Although visual field testing is commonly used in adults (Johnson, 2002; Kedar et al., 2011), there are limited studies about visual field testing on children. This may in part be attributed to the challenges associated with obtaining reliable test results (Walters et al., 2012; Billson, 2000). Children may not be able to meet the minimum test standard as adults since they have shorter attention spans and can get distracted easily during the test. Extra encouragement and instructions are needed for children to complete a reliable visual field test. However, the ability to detect peripheral targets within the visual field should not be neglected in children. Visual sensitivity is an important factor for children to detect peripheral targets, especially in sports performance (Popowczak et al., 2020; Kung et al., 2020). It not only enhances their performance in sport but it also reduces the injury risk if good visual sensitivity can be obtained.

This is the first study using static perimetry to investigate the visual field performance using the DIMS lens and comparing to a conventional SV lens in children. A study investigated the visual sensitivity between the H.A.L.T. technology lens (Stellest), a concentric ring design with aspheric lenslets for myopic control lens and compared this to a conventional SV lens in adults (Gao et al., 2022). They found no significant differences in the visual sensitivity to static targets within 30-degree eccentricity between the Stellest and SV lens. The authors suggested that this was attributed to the concentric lenslets accounting for 40% of the lens area and 60% of the test lens area accounted for by a clear zone. Although the design of defocus power in DIMS lens was uniform and fixed (+3.5 D), our result showed that there were no significant differences in visual sensitivity when compared to SV lens. Several studies (Wu et al., 2024; Gao et al., 2023) have demonstrated peripheral visual performance using myopia control lenses. Compared to the spectacle lenses with highly aspherical lenslets (HAL) and orthokeratology lenses (OK lenses), it has been demonstrated HAL lense increased the peripheral coherence threshold for identifying the contraction movement than OK lenses, and they recommended that OK lenses are better for children when there is more specific global scene recognition and movement requirements (Wu et al., 2024). Compared with SV lenses, the HAL lens reduced high-contrast central VA with peripheral gaze at low luminance and such reduction in peripheral VA was not relevant to the peripheral gaze direction (Gao et al., 2023). In this study, there was a decrease in mid-peripheral NVA (about 0.05 logMAR) using DIMS lenses compared to SV lenses (Figure 6). However, the correlation between the mid-peripheral NVA and visual sensitivity in SV and DIMS lenses group was found insignificant (Tables 4, 5).

Previous studies (Lam et al., 2020a; Lu et al., 2019; Kaymak et al., 2022; Li et al., 2021) showed that DIMS lens had little or no impact on visual performances, including visual acuity at distance and near, accommodation, stereopsis and horizontal phoria. However, there was a slight reduction (Approximately 0.10 logMAR) in peripheral visual acuity (off-axis) in children when comparing different myopia control lenses such as DIMS and Stellest to a conventional SV lens (Li et al., 2021). Yet, there were no significant differences in central visual acuity (on-axis) between DIMS and SV lens (Lu et al., 2019). Also, it was found that the mid-peripheral NVA was reduced about 0.06 logMAR compared to SV lens (Lu et al., 2019). Such blur may lead to drop in contrast sensitivity. In this study, we found the mid-peripheral NVA using DIMS lens was decreased about 0.05 logMAR compared to SV lens. Although it was statistically significant (Figure 6), such decrease may not be clinically significant as it was about half of the line. Children may notice the blur in the peripheral part during their daily activities such as reading and using laptop. They may need to adjust their posture in order to optimize their vision.

It was reported that the DIMS lens had significantly reduced overall contrast sensitivity, especially at high spatial frequencies (12 and 18 cycles per degree) compared to SV lens at each luminance levels, with and without glare disturbance (Li et al., 2021). Surprisingly, the reductions in contrast sensitivity in DIMS lens compared to SV lens at low spatial frequencies (3 and 6 cycles per degree) was less significant. DIMS lens may produce a lower contrast image at high spatial frequency and generate a high contrast image if the spatial frequency is smaller than 7 cycles per degree (Jaskulski et al., 2020). The concentric rings with aspherical lenslets (Stellest) had significantly lower impact on contrast sensitivity than honeycomb configurations defocus design (DIMS) (Li et al., 2021) while Stellest lens had similar visual sensitivity compared with SV lens (Gao et al., 2022). It is therefore questionable whether the reduction in contrast sensitivity in the DIMS lens would impact the visual field. In the current study, although the difference in visual sensitivity between DIMS and SV lens ranged from −2.4 ± 3.9 dB to 1.6 ± 3.9 dB, the differences were not statistically significant. It may also explain why the lens performance in vision stability, ease of lens adaption and overall performance was similar between DIMS and SV lens (Lam et al., 2020a). Additionally, there was no significant correlation between visual sensitivity and mid-peripheral NVA between the DIMS and SV lens. This suggests that the slight drop in off-axis visual acuity does not impair the peripheral visual field.

The static perimeter utilized adult-based normative data such as Mean Deviation (MD) and Pattern Standard Deviation (PSD) to measure the performance. The machine itself did not contain child-based normative data. In order to compare the visual performance in children between DIMS and SV lens, visual sensitivity was used. In this study, the overall mean visual sensitivity was similar between DIMS (29.2 ± 3.7 dB) and SV (29.3 ± 3.5 dB) lens. There was no significant difference in visual sensitivity between DIMS and SV lens at each of the 76 locations within a 30-degree. This suggests that the defocus power in DIMS lens does not decrease the ability to detect peripheral targets in children significantly. Although there was no statistically significant difference in visual sensitivity in all positions and most of them showed no practically significant between DIMS and SV lenses, we did observe a considerable difference in visual sensitivity at row 2a was 2.4 dB (Figure 7c), indicating that DIMS wearers need 74% brighter of the light to detect. Yet, the row 2a was further than the superior-nasal 20-degree, which may not lead to a significant impact on daily life. Nevertheless, a larger sample size is warranted to investigate the potential impact.

There are some limitations in the current study. Firstly, we used static perimetry instead of kinetic perimetry to measure the visual performance in children. In real life, the stimulus will not always be stationary. Kinetic visual field tests may be more suitable to assess the “real” visual performance in children. However, kinetic perimetry cannot be performed with the trial lens ring in place as this may produce rim artifact. A future study that investigates the kinetic sensitivity of the DIMS lenses is warranted. Additionally, a 30-degree visual field and short viewing distance (30 cm) was evaluated in the current study using the DIMS lens. The visual field in real life is not limited to 30-degree. Therefore, further studies are needed to test the sensitivity with higher eccentricity and longer working distance.

Secondly, the age of the participants in the present study may be considered as older children. A previous similar study used an adult population when testing and comparing the sensitivity of the Stellest lens to a conventional SV lens (McKean-Cowdin et al., 2007). This is the first study to investigate the visual field performance using the DIMS lens in children. Given the challenges in performing visual field assessment in adult subjects and the older children in the present study, it is likely that measuring visual performance using a visual field test would be even more challenging in younger kids due to shorter attention spans. Perhaps further studies may be able to overcome some of these challenges by using a shorter, computer-based game that is while testing the visual field, making it interactive for children (Miranda et al., 2016). Thirdly, the sample size of the current study may not be large enough to generalize the results across the population. Future study may require more children with different ages, especially younger kids, to be participated in the evaluation of the peripheral visual performance using myopic control lenses.

## Conclusion

In conclusion, our findings provide information about the static automated visual field values in children using the DIMS and SV lenses despite the reduced mid-peripheral near VA in DIMS lens compared to SV lens. Most subjects performed well on the visual field assessment suggesting that both lenses have similar visual field sensitivity. Evaluation of the visual sensitivity on children using DIMS lenses will assist practitioners to understand the design of the myopic control lenses. It also enhances the communication between clinicians, children and their parent about the usage and possible side effects using the DIMS spectacle lenses. On the other hand, it is worthy to study the long term effect of the peripheral visual performance using DIMS spectacle lenses.

## Data Availability

The raw data supporting the conclusions of this article will be made available by the authors, without undue reservation.
